# Supplementary material for: Distribution of deep-water scleractinian and stylasterid corals across abiotic environmental gradients on three seamounts in the Anegada Passage
Source: PeerJ. 2020 Jul 31;8:e9523. doi: 10.7717/peerj.9523 (PMC7397984; doi:10.7717/peerj.9523)

Supplementary Figure 1: Aragonite saturation state profiles for the Anegada Passage using measured (closed circle) and model predicted (open circle) Ω_{arag} values (see Supplementary Table 1). A solid red vertical line at Ω_{arag} = 1 represents the aragonite saturation horizon.

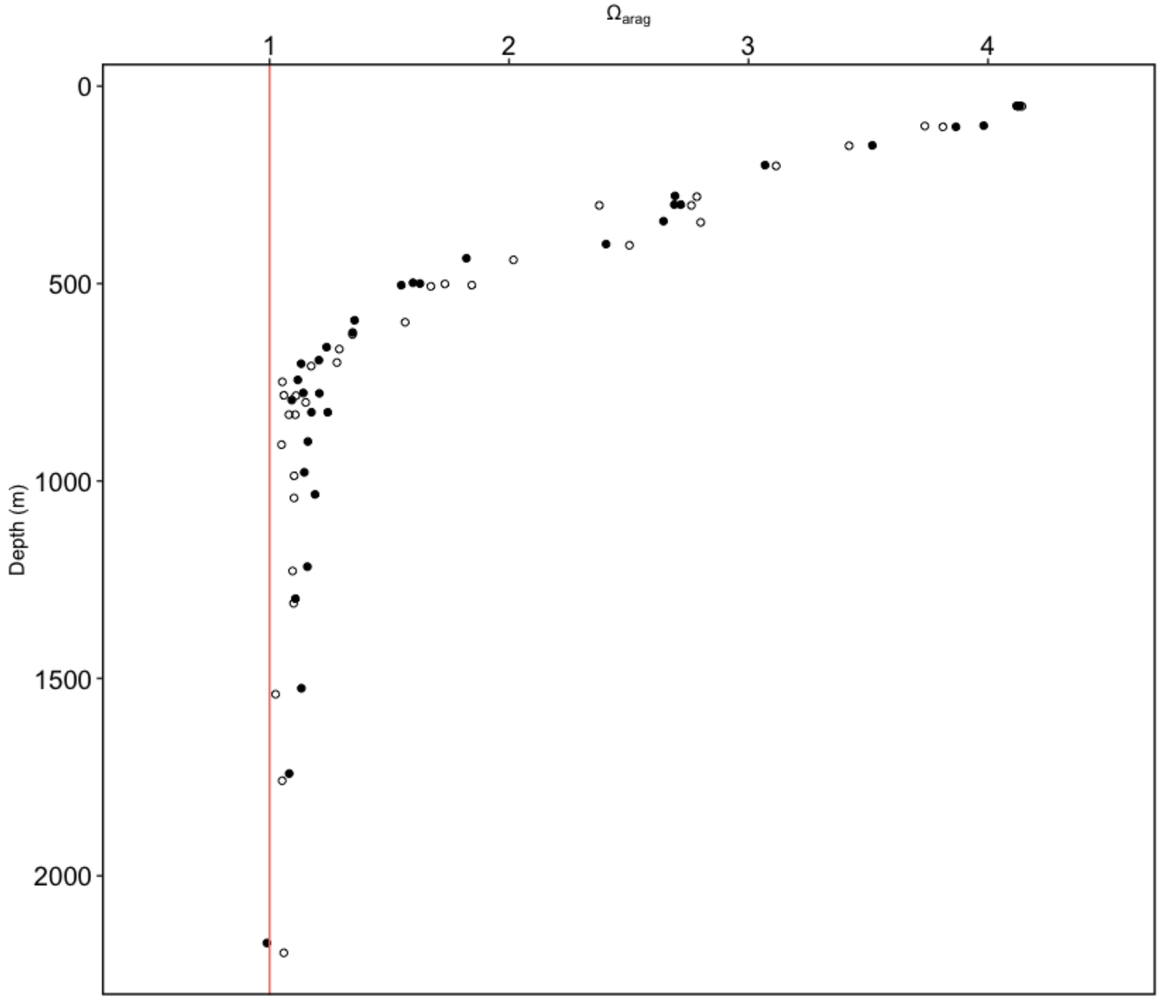

Supplement: Supplemental Information 1 — A solid red vertical line at Ωarag = 1 represents the aragonite saturation horizon. [file peerj-08-9523-s001.pdf]
