# Supplementary material for: Distribution of deep-water scleractinian and stylasterid corals across abiotic environmental gradients on three seamounts in the Anegada Passage
Source: PeerJ. 2020 Jul 31;8:e9523. doi: 10.7717/peerj.9523 (PMC7397984; doi:10.7717/peerj.9523)
Supplement: Supplemental Information 3 — Indicated values are R-statistics with p-values indicated in parentheses. Values in bold were observed to be significant at or below p=0.05. [file peerj-08-9523-s003.docx]

Supplementary Table 2: Results of the two-way nested ANOSIM (depth within seamount) comparing coral assemblages between seamounts. Indicated values are R-statistics with p-values indicated in parentheses. Values in bold were observed to be significant at or below p=0.05.

| Seamount | Conrad Seamount | Dog Seamount | Noroît Seamount |
| --- | --- | --- | --- |
| Conrad Seamount |  |  |  |
| Dog Seamount | -0.001  (0.379) |  |  |
| Noroît Seamount | 0.029  (0.292) | **0.544**  **(0.006)** |  |
