# Supplementary material for: Distribution of deep-water scleractinian and stylasterid corals across abiotic environmental gradients on three seamounts in the Anegada Passage
Source: PeerJ. 2020 Jul 31;8:e9523. doi: 10.7717/peerj.9523 (PMC7397984; doi:10.7717/peerj.9523)
Supplement: Supplemental Information 4 — Indicated values are R-statistics with p-values indicated in parentheses. Values in bold were observed to be significant at or below p=0.05. Water masses are abbreviated by the following: SUW=Subtropical underwater, SSW=Sargasso Sea Water, TACW= Tropical Atlantic Central Water, AAIW=Antarctic Intermediate Water, and NADW=North Atlantic Deep Water. [file peerj-08-9523-s004.docx]

Supplementary Table 3: Results of a one-way ANOSIM comparing coral assemblages between water mass factors. Indicated values are R-statistics with p-values indicated in parentheses. Values in bold were observed to be significant at or below p=0.05. Water masses are abbreviated by the following: SUW=Subtropical underwater, SSW=Sargasso Sea Water, TACW= Tropical Atlantic Central Water, AAIW=Antarctic Intermediate Water, and NADW=North Atlantic Deep Water.

| Water Mass | SUW | SSW | TACW | AAIW | NADW |
| --- | --- | --- | --- | --- | --- |
| SUW |  |  |  |  |  |
| SSW | 0.16  (0.167) |  |  |  |  |
| TACW | 1.0  (0.125) | **0.611**  **(0.003)** |  |  |  |
| AAIW | 0.667  (0.091) | **0.530**  **(0.002)** | 0.089  (0.134) |  |  |
| NADW | 0.8  (0.167) | **0.6**  **(0.008)** | **0.93**  **(0.001)** | **0.361**  **(0.01)** |  |
